# Genomic responses to hepatitis B virus (HBV) infection in primary human hepatocytes

**DOI:** 10.18632/oncotarget.6270

**Published:** 2015-11-02

**Authors:** Pierre-Benoit Ancey, Barbara Testoni, Marion Gruffaz, Marie-Pierre Cros, Geoffroy Durand, Florence Le Calvez-Kelm, David Durantel, Zdenko Herceg, Hector Hernandez-Vargas

**Affiliations:** ^1^ Epigenetics Group, International Agency for Research on Cancer (IARC), Lyon, France; ^2^ INSERM U1052, Molecular Physiopathology and New Treatments of Viral Hepatitis, Centre de Recherche en Cancérologie (CRCL), Lyon, France; ^3^ Genetic Cancer Susceptibility Group, International Agency for Research on Cancer (IARC), Lyon, France

**Keywords:** HBV, hepatitis, methylome, HM450, transcriptome

## Abstract

Viral infections are able to modify the host's cellular programs, with DNA methylation being a biological intermediate in this process. The extent to which viral infections deregulate gene expression and DNA methylation is not fully understood. In the case of Hepatitis B virus (HBV), there is evidence for an interaction between viral proteins and the host DNA methylation machinery. We studied the ability of HBV to modify the host transcriptome and methylome, using naturally infected primary human hepatocytes to better mimic the clinical setting.

Gene expression was especially sensitive to culture conditions, independently of HBV infection. However, we identified non-random changes in gene expression and DNA methylation occurring specifically upon HBV infection. There was little correlation between expression and methylation changes, with transcriptome being a more sensitive marker of time-dependent changes induced by HBV. In contrast, a set of differentially methylated sites appeared early and were stable across the time course experiment. Finally, HBV-induced DNA methylation changes were defined by a specific chromatin context characterized by CpG-poor regions outside of gene promoters.

These data support the ability of HBV to modulate host cell expression and methylation programs. In addition, it may serve as a reference for studies addressing the genome-wide consequences of HBV infection in human hepatocytes.

## INTRODUCTION

Methyl residues in cytosines are able to translate environmental exposures into cellular phenotypes [1, 2]. Such DNA methylation marks can be transmitted through cell division, and contribute to the establishment of defined traits, including disease susceptibility [1, 2]. Although DNA methylation is known to change throughout human lifetime, there is little information on the specific environmental exposures responsible for those changes. At the genomic level, there is no consensus on the locations or DNA/chromatin contexts susceptible to that modulation.

Viral infections are known to affect the host methylome [3]. In the case of Hepatitis B virus (HBV), there is evidence for a direct interaction between HBV X protein (HBx) and human DNA methyl-transferases (DNMTs) upon infection of host hepatocytes [4–9]. HBV is a well known risk factor for several chronic liver pathologies, such as hepatitis, cirrhosis, and cancer [10]. In all of these conditions, aberrant DNA methylation has been described for the targeted hepatocytes. For example, data on clinical samples has shown that HBV-related hepatocellular carcinoma (HCC) displays a specific DNA methylation profile [11, 12]. However, the extent to which this signature is differentially contributed by HBV infection and the secondary chronic inflammatory response is not known. In a similar way, it is not clear whether HBV-induced changes in methylation are an early consequence of infection.

In the present report we studied the extent to which HBV infection affects the host transcriptome and methylome. To this end, we took advantage of a physiological model of natural HBV infection in primary human hepatocytes (PHH) and genome wide tools. We further studied the association between methylation and transcriptional changes.

## RESULTS

### Dynamic changes in gene expression in cultured primary hepatocytes

**Figure 1:**
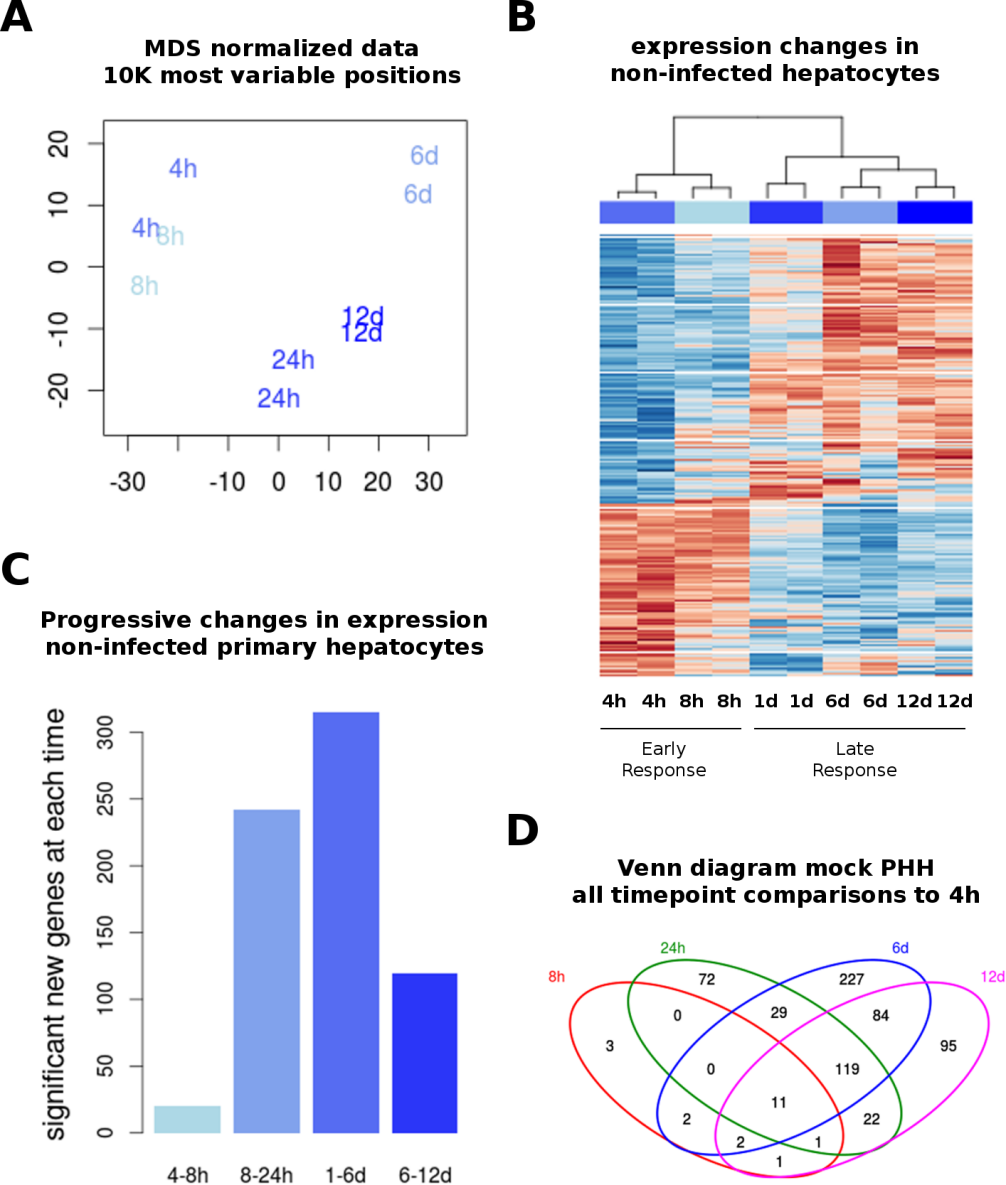
Transcriptome in non-infected PHH Whole genome expression analysis was performed on primary human hepatocytes (PHH) after several time points in culture (as described in Materials and Methods). (**A**) Multidimensional scaling (MDS) representation of expression distances, representing duplicates of each time point used for all gene expression analyses. Time points are indicated in text and colors. (**B**) Differential expression was performed by comparing samples from each time point to the earliest (4 hours) time point in non-infected cells. All differentially expressed genes (FDR < 0.05, fold-change > 2) were used to plot an unsupervised heatmap in a blue-red scale (low to high expression). Samples cluster by replicate and time point. (**C**) Pair-wise comparisons were done between immediate time points to investigate the progressive changes in gene expression. (**D**) Overlap between differentially expressed genes (using gene symbols) from the analysis shown in (B), displayed as Venn diagram.

Cultured primary human hepatocytes (PHH) are the closest *in vitro* model to human liver and constitute a very predictive model for pharmaco-toxicology *in vivo* [13]. To understand the effect of cell culture on hepatocyte cellular programs, we studied the dynamics of gene expression at different time points after plating PHH. RNA extracted at different time points from duplicated wells from one single donor, was processed for whole genome expression arrays. Unsupervised analyses were used to assess the relative distance among the different conditions. Replicates from each condition clustered together at all time points (Figure 1A and 1B). To study the dynamics of these changes, we compared the gene expression profiles at each time point to the earliest time point (1 day in culture + 4 hours post-mock treatment). Clustering of the samples using all significant genes at each time point (FDR < 0.05, Fold Change ≥ 2) divides the conditions into early (before than 24 h) and late (24 h and later) responses (Figure 1B). Gene expression changes were cumulative, with up to 540 differentially expressed genes by day 6, and a slight decay at day 12. However, most of the changes occurring after 24 h did not overlap with early gene expression differences (Figure 1C and 1D). In addition, the largest changes between two adjacent time points occurred at 24 h and 6 days of culture with 242 and 315 differentially expressed genes, respectively (Figure 1C). At day 12 there were relatively less expression changes, suggesting some type of adaptation.

To get an insight into the identity of these dynamically differentially expressed genes in non-infected cells, we studied their enrichment in known functional pathways (using an adjusted *P* value < 0.05 and at least 3 matching genes as criteria). Taking together all differentially expressed genes across all time points, we found a highly significant enrichment in HNF4A targets, using the ChEA database (overlap of 1045 differentially expressed genes to 6083 genes in the HNF4 dataset) (Table S2). Samples obtained at 24 hours displayed the most unique expression profile, with a significant enrichment in proteasome degradation factors as assessed by several datasets (i.e. KEGG, WikiPathways, Reactome, and BioCarta).

In summary, *ex-vivo* culture has strong effects on PHH gene expression profiles. These changes are time-dependent and enriched in HNF4A targets, a well-known master transcription factor of hepatocyte differentiation [14, 15].

### HBV infection induces time-dependent changes in gene expression

**Figure 2:**
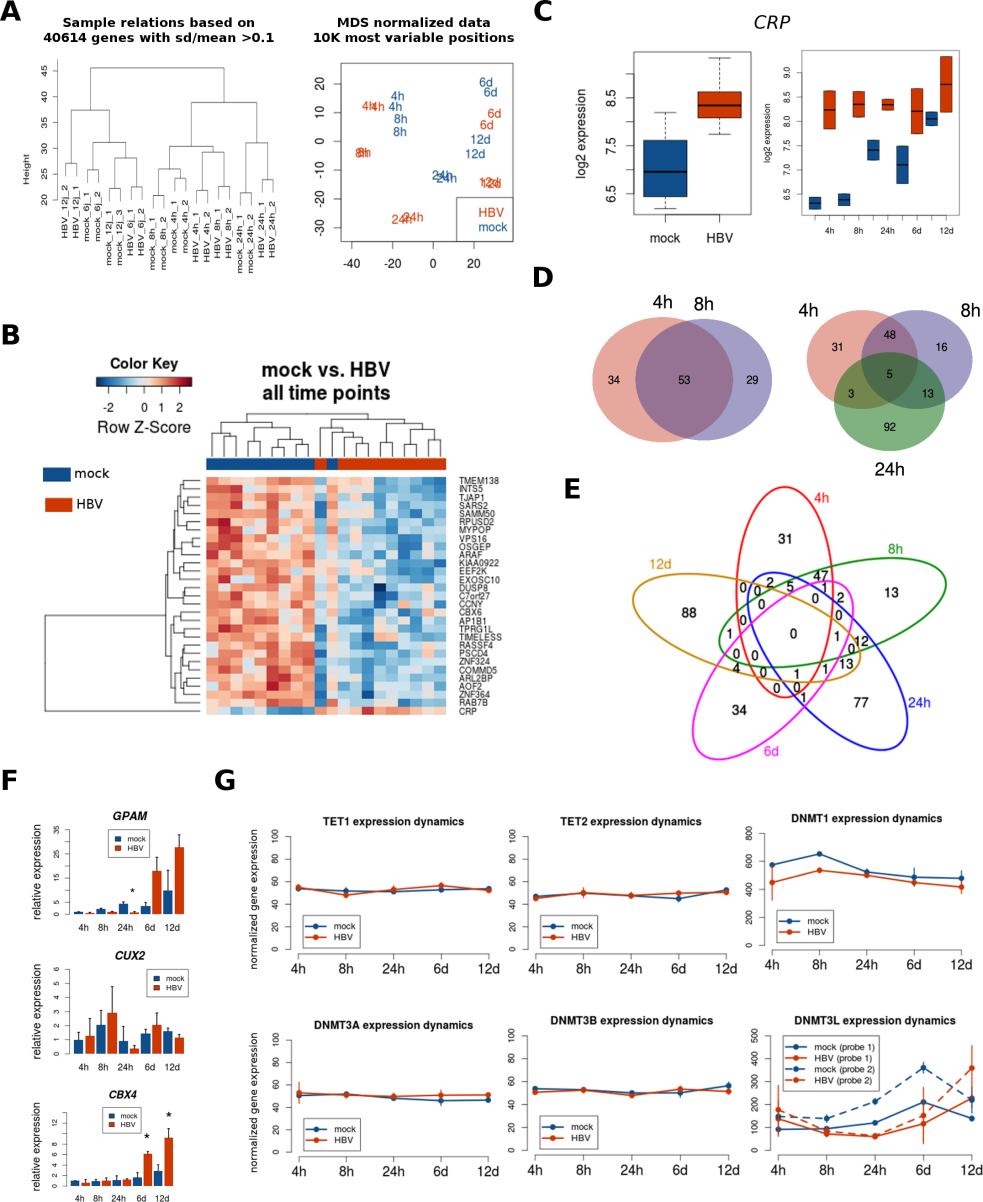
Transcriptome analysis after HBV infection PHH were naturally infected with HBV during different time points and processed for whole genome expression using Illumina bead arrays, as described in Materials and Methods. (**A**) Sample relationships using unsupervised clustering (left panel) and multidimensional scaling (right panel) of all gene expression data shows that samples cluster successively by time point, infection condition (mock vs. HBV) and replicate. (**B**) Heatmap of genes differentially expressed between mock- and HBV-infected PHH (FDR < 0.05, no fold-change criteria) across all time points. (**C**) Expression plot of one differentially expressed gene (CRP) using the mean of all time points (left panel), or the means for each time point separately (right panel). (**D**) Overlap between HBV differentially expressed genes at early time points (FDR < 0.05, fold-change > 2). (**E**) Overlap between HBV differentially expressed genes at all time points (FDR < 0.05, fold-change > 2). (**F**) Validation of selected genes using qRT-PCR. Asterisk represents statistical significance at the indicated time point (*P* < 0.05). (**G**) Gene expression for DNA-methylation (and demethylation) players was extracted from whole genome expression data, and their means for each time point were plotted separately for mock- and HBV-infected PHH. For DNMT3L two independent probes are represented.

To determine the potential ability of HBV to induce gene expression changes in the host cell, we performed a natural infection of PHH during the same time points described above and analyzed their whole-genome expression. PHH kept on culture for the same time points were used as controls. Natural infection was highly effective, as illustrated by the expression of viral proteins (Figure S1A and S1B). Time in culture was the strongest component of variation in gene expression. However, for each time point HBV-infected hepatocytes clustered apart from mock-treated PHH (Figure 2A and S1C). In agreement with this, only 29 genes were differentially expressed in HBV conditions across all time points without considering the fold-change (Figure 2B and Table 1). However, only one of those genes (the C reactive protein coding gene, *CRP*) displayed an overall change of more than 2-fold across all time points, although this difference was especially evident at early time points (Figure 2C). Although there was a significant overlap between the differentially expressed genes at 4 and 8 hours (Figure 2D – left panel), all other time points of HBV infection were associated with mainly unique differences in gene expression (using FDR < 0.05 and minimum fold-change of 2 as criteria) (Figure 2D – right panel and 2E, and Table S2). A random selection of differentially expressed genes was technically validated by qRT-PCR (Figure 2F). Of note, similar results were obtained in an independent PHH preparation from an unrelated donor (Figure S2A).

**Table 1:** Genes differentially expressed in response to HBV infection across all time points in primary human hepatocytes

Symbol	FC	FDR
*TJAP1*	0.74	0.00
*RPUSD2*	0.79	0.01
*ARL2BP*	0.76	0.02
*RAB7B*	0.76	0.02
*ZNF324*	0.84	0.02
*ZNF364*	0.71	0.02
*COMMD5*	0.82	0.02
*ARAF*	0.8	0.02
*KIAA0922*	0.78	0.02
*OSGEP*	0.81	0.02
*AOF2*	0.8	0.02
*AP1B1*	0.81	0.02
*SARS2*	0.8	0.02
*CCNY*	0.74	0.02
*EXOSC10*	0.81	0.02
*VPS16*	0.77	0.03
*DUSP8*	0.69	0.03
*TPRG1L*	0.8	0.03
*MYPOP*	0.8	0.04
*CRP*	2.51	0.04
*SAMM50*	0.83	0.04
*PSCD4*	0.82	0.04
*TMEM138*	0.82	0.05
*TIMELESS*	0.82	0.05
*C7orf27*	0.74	0.05
*CBX6*	0.77	0.05
*RASSF4*	0.86	0.05
*INTS5*	0.74	0.05
*EEF2K*	0.76	0.05

FC = fold change (HBV/mock). FDR = false discovery rate

At all time points, HBV-induced changes in gene expression were enriched in liver-related genes, as assessed with the Human Gene Atlas database. Top enriched pathways at early time points (4 and 8 hours) included the Interferon Type I signaling pathway, and several metabolic pathways such as bile acid biosynthesis, tyrosine and glycerolipid metabolism (Table S2). This deregulation in metabolic pathways was much more evident at 24 hours of HBV infection with enrichment in multiple liver-related signatures including glycolysis, fatty acid metabolism, and one carbon metabolism (Table S2). Indeed, differentially expressed genes at 24 h of HBV infection were highly enriched in known HNF4 targets (Table S2). Therefore, HBV infection induces specific changes in gene expression that are independent from the time-related changes due to PHH cell culture.

Previous studies have reported an association between HBV infection and differential expression of certain DNA methylation players, such as the *de novo* methyltransferases DNMT3A and DNMT3B [4–9]. Therefore, as a baseline for interpreting DNA methylation results, we extracted the gene expression data corresponding to genes involved in DNA methylation (DNMTs) and demethylation (TETs). As shown in Figure 2G, only *DNMT3L* displays differential expression upon HBV infection (statistically significant at 24 h post-infection).

### Region-level differential DNA methylation in response to HBV infection

**Figure 3:**
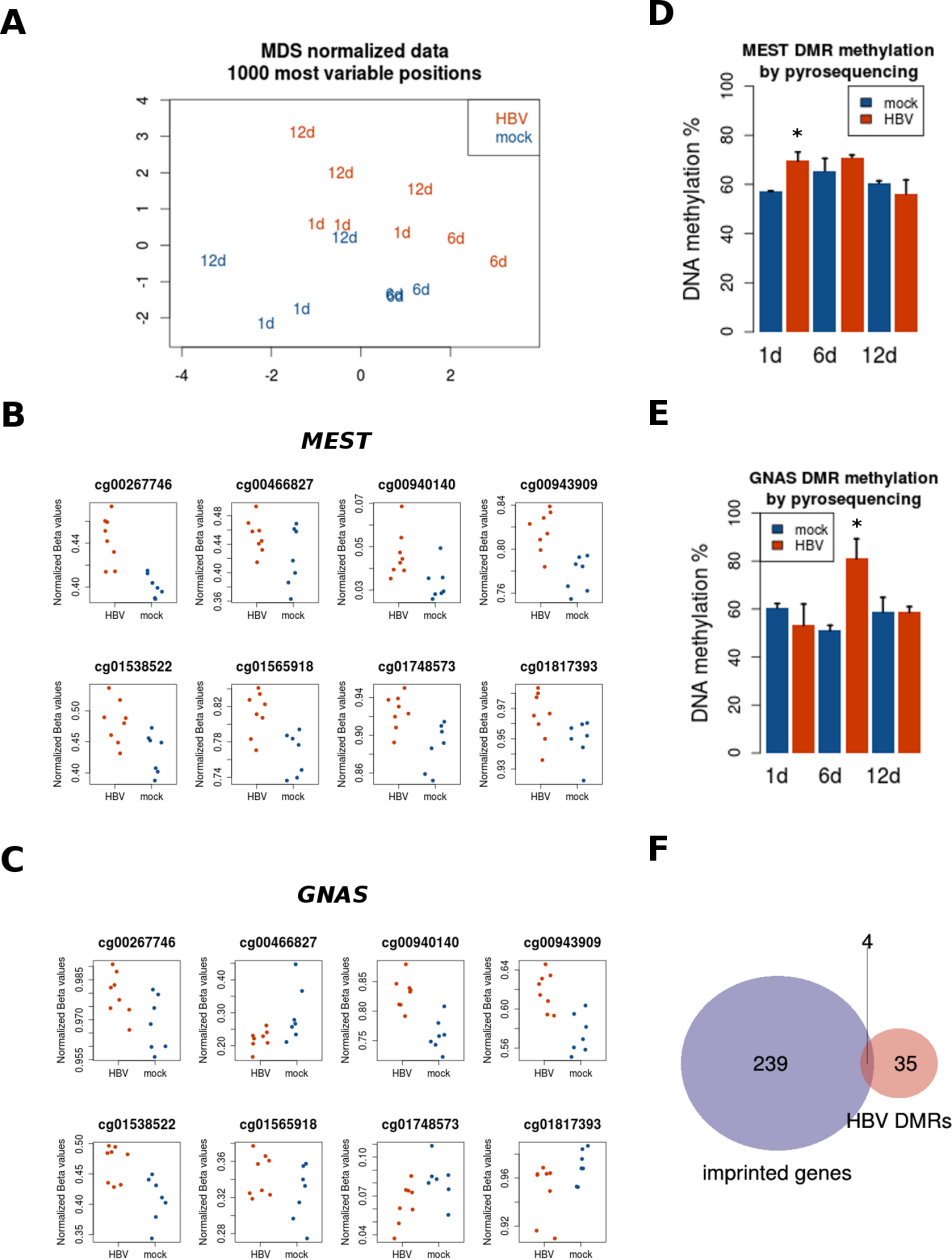
Region-level DNA methylation changes induced by HBV PHH were treated with the same conditions described above for gene expression analysis, and collected after several time points (1, 6, and 12 days). DNA was extracted and processed for genome-wide DNA methylation using Illumina Infinium 450K arrays. (**A**) MDS plot of most variable methylation sites shows replicates clustering together, and global differences in methylation between mock- and HBV-infected PHH. Regional differences in DNA methylation were assessed as described in Materials and Methods. Two of the top differentially methylated regions (DMRs) are shown: *MEST* (**B**) and *GNAS* (**C**), as well as their corresponding validations by bisulfite pyrosequencing (**D** and **E**, respectively). (**F**) The overlap between DMRs and known imprinted genes is represented in the Venn diagram.

To determine the potential ability of HBV to induce DNA methylation changes in the host cell we performed a natural infection of PHH during 1, 6, and 12 days. PHH kept on culture for the same time points were used as controls. DNA obtained from the different conditions was bisulfite modified and studied for DNA methylation using the HM450 bead arrays (as described in Materials and Methods). Of note, DNA used for genome-wide methylation analyses and RNA used for transcriptome analyses were extracted from the same PHH donor. As opposed to gene expression data, DNA methylation was able to distinguish HBV-exposed from control PHH, regardless of the time in culture (Figure 3A).

It has been shown that CpG sites within the same CpG island tend to behave in a coordinated manner [16]. We used this property of DNA methylation to study regional changes induced by HBV and identify so called differentially methylated regions (DMRs). Taking together all time points, there were no significant DMRs distinguishing HBV- from mock-treated PHH. However, when analyzing separately each time point, we found 42 DMRs after one day of infection (adjusted *P* value < 0.05, and at least two CpGs per region) (Table 2). Of note, some of the top most significant DMRs are known imprinted genes such as *MEST* and *GNAS* (Figure 3B and 3C, respectively). Several CpG sites (13 for *MES*T and 11 for *GNAS*) displayed an increased methylation after HBV exposure (Figure 3B and 3C, respectively). No DMRs were found at 6 and 12 days post-infection. Validation by pyrosequencing confirmed the HBV-related hypermethylation in *MEST* (including *MEST* imprinted control region –ICR- [Figure S2B]) and *GNAS* loci, although for this last locus the highest change was observed at the 6 days-time point, instead of the expected difference at 24 hours based on the bead array data (Figure 3D and 3E, respectively).

**Table 2:** Differentially methylated regions (DMRs) after one day of HBV infection of primary human hepatocytes

Genomic location	Symbol	CpGs	Promoter
chr7:130131869–130132286	*MEST*	13	TRUE
chr6:144329052–144329485	*PLAGL1*	6	FALSE
chr20:57463783–57463925	*GNAS*	6	FALSE
chr11:10315609–10315761	*SBF2*	5	TRUE
chr5:14871736–14871910	*ANKH*	5	TRUE
chr8:74207183–74207587	*RDH10*	5	FALSE
chr22:24890794–24890831	*UPB1*	5	FALSE
chr6:2765585–2765945	*WRNIP1*	4	TRUE
chr11:2160540–2160564	*IGF2*	4	FALSE
chr11:3688526–3689006	*CHRNA10*	4	FALSE
chr17:80477464–80477962	*FOXK2*	4	TRUE
chr17:42297002–42297053	*UBTF*	4	TRUE
chr21:45138838–45139229	*PDXK*	4	TRUE
chr16:4897378–4897921	*UBN1*	4	FALSE
chr11:77907332–77908054	*USP35*	3	TRUE
chr15:63340581–63340702	*TPM1*	3	FALSE
chr16:67063319–67063591	*CBFB*	3	FALSE
chr3:126260615–126261298	*CHST13*	3	TRUE
chr16:68056778–68056948	*DUS2*	3	FALSE
chr17:36717733–36718549	*SRCIN1*	3	FALSE
chr10:112257641–112257943	*DUSP5*	3	FALSE
chr5:76373091–76373719	*ZBED3*	3	FALSE
chrX:41332957–41333643	*NYX*	3	TRUE
chr2:205410108–205410387	*PARD3B*	3	FALSE
chr20:57465439–57465448	*GNAS*	3	FALSE
chr12:111843885–111843939	*SH2B3*	3	FALSE
chr7:6692445–6692873	*ZNF853*	2	FALSE
chr2:150186921–150186923	*LYPD6*	2	FALSE
chr16:73092391–73092394	*ZFHX3*	2	FALSE
chr2:111880006–111880018	*BCL2L11*	2	FALSE
chr19:11071743–11071746	*SMARCA4*	2	TRUE
chr21:47706156–47706161	*YBEY*	2	FALSE
chr19:51601884–51602230	*CTU1*	2	TRUE
chr8:102218219–102218365	*ZNF706*	2	FALSE
chr11:66624256–66624258	*PC*	2	FALSE
chr8:144810034–144810339	*FAM83H*	2	FALSE
chr20:57464970–57464973	*GNAS*	2	FALSE
chr19:51607432–51607839	*CTU1*	2	FALSE
chr15:74315331–74315474	*PML*	2	FALSE
chr7:149321876–149321879	*ZNF767P*	2	FALSE
chr22:22652529–22652537	*BMS1P20*	2	FALSE
chr19:1228888–1229184	*STK11*	2	FALSE

Imprinted genes are mono-allelically expressed in a parent-of-origin manner, a process tightly controlled by DNA methylation [17]. Because of the potential interest in their response to a diversity of exposures, we studied the enrichment of known imprinted genes in our list of DMRs at 24 h of HBV infection. Only 4 genes (*GNAS, IGF2, MEST*, and *PLAGL1*) overlapped between the 42 DMRs (corresponding to 39 unique gene symbols) and a list of 243 known imprinted genes (Figure 3F). Although small, this overlap was higher than expected by chance (representation factor = 8.4, *P* < 0.001). Therefore, certain imprinted loci seem to be sensitive to HBV exposure. This is in line with the lower expression of *DNMT3L* (Figure 2G), a cofactor of *de novo* DNA methyl-transferases in methylation of imprinted loci [18].

### Site-specific differential DNA methylation in response to HBV infection

**Figure 4:**
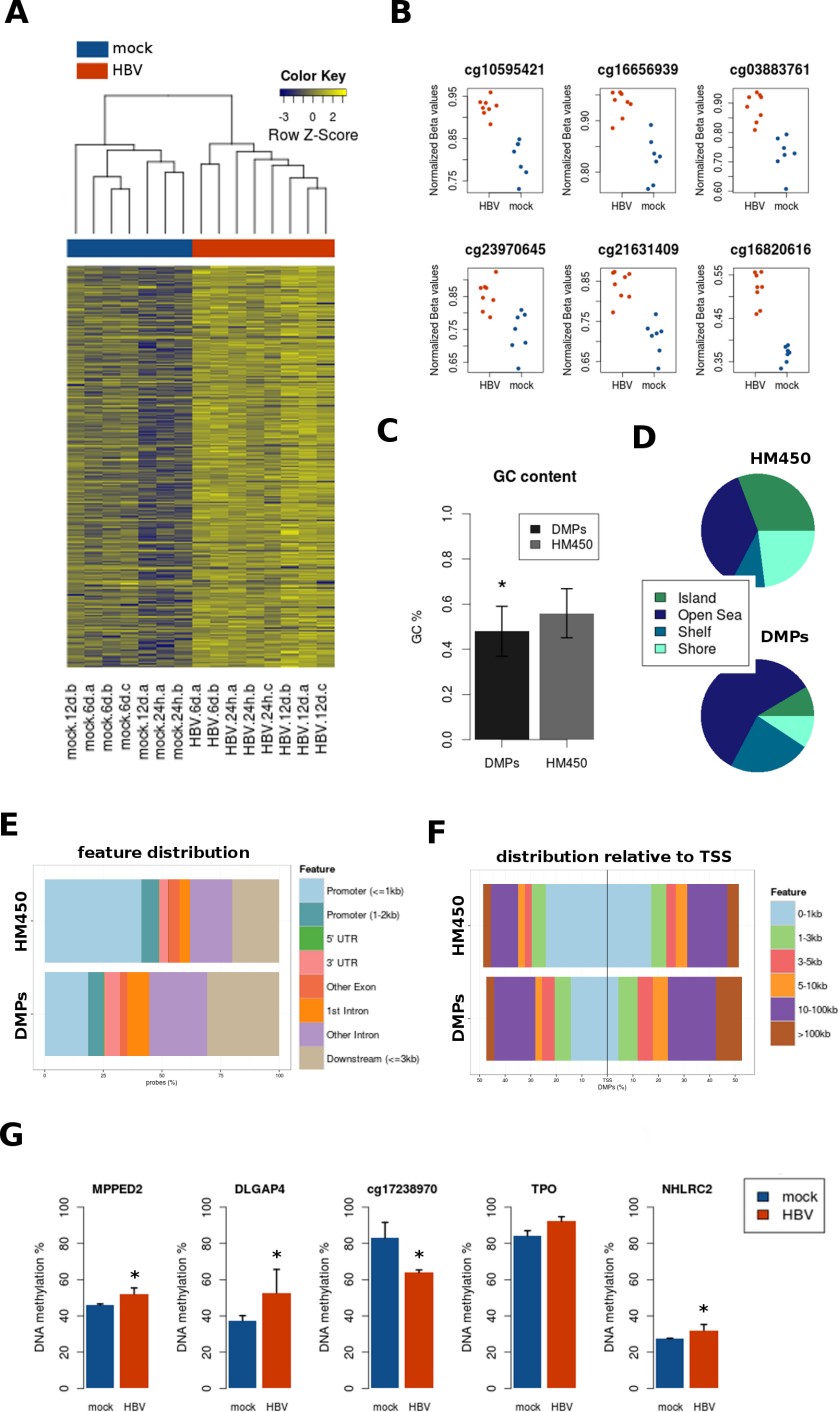
Site-level DNA methylation changes induced by HBV (**A**) Heatmap of differentially methylated positions (DMPs) between all mock and all HBV samples, regardless of time point (FDR < 0.05, delta_beta ≥ 10%). DNA methylation is represented in a blue-yellow scale, from lower to higher methylation. (**B**) The top CpG sites (with lower *p* values) are presented in a dot plot comparing mock (blue) and HBV (red) conditions, with normalized beta values on the y axis. (**C**) GC content was significantly lower on DMPs (*p* < 0.05), as compared to the whole content of Illumina 450 k probes (HM450). (**D**, **E**, and **F**) Distribution of DMPs was analyzed according to CpG islands (shores, shelves, islands, and “open sea”) (**D**), position relative to genes (promoter, UTRs, intron/exon) (**E**), and distance to transcription start sites (TSS) (**F**) Total bead array probe distribution (HM450) is shown for all plots as a reference. (**G**) Bisulfite pyrosequencing validation of a random selection of DMPs.

We next studied site-by-site differences in DNA methylation, as this accounts for CpG sites in CpG poor regions of the genome. Although no differential methylation was found when comparing HBV-treated and control PHH samples at each specific time point, we found 287 differentially methylated positions (DMPs) when taking all time points simultaneously (Figure 4A, and Table S2). Most of these sites were hypermethylated after HBV infection (*n* = 258), relative to mock-treated samples (FDR < 0.05, change in methylation of at least 10%) (Figure 4A and 4B). DMP probes were characterized by a lower GC content, compared to the total of HM450 probes (Figure 4C). This was consistent with a relative absence of DMPs from CpG islands (Figure 4D). Instead, DMPs tended to accumulate in the so called “open sea” and intronic regions, and far from promoters and transcription start sites (TSS) (Figure 4D, 4E and 4F). A random selection of DMPs was correctly validated by quantitative bisulfite pyrosequencing (Figure 4G). However, differences in methylation were not observed after HBV infection of PHH obtained from an independent donor (Figure S2B). Finally, DMPs in this analysis did not overlap with the differentially methylated regions (DMRs) described above, probably because of their lower magnitude of change (less than 10% difference).

Performing pathway/ontology analysis in HM450 data may result in spurious associations due to the unbalanced representation of probes for different genes within the array [19]. To overcome this issue, we adjusted for the number of probes per gene symbol and selected only those genes with at least one significant CpG site below the FDR-ajusted *P* value threshold of 0.05 (see Materials and Methods). The resulting 125 gene symbols were used for pathway enrichment analyses using the Enrichr gene list enrichment web tool (Table S2). Gap junction and axon guidance were the top most significant pathways in three different databases (i.e. KEGG, WikiPathways and Reactome), with DMPs found in 7 genes belonging to these pathways (i.e. *SRC, HTR2C, EGFR, PRKG1, CREB1, UNC5A*, and *KCNQ2*). In addition, the top most significant pathway using BioCarta was “calcium signaling by HBx of hepatitis b virus”, and it included two genes from the previous list (i.e. *SRC* and *CREB1*) (Table S2).

Together, these data show that DNA methylation is sensitive to unbiased changes upon HBV infection in PHH. Although less variable than gene expression (in terms of number of significant associations), DNA hypo and hypermethylation were consistent across all time points for a subset of CpG sites. Although HBV-induced DMPs seem to be specific of each liver donor (and consequently, of each PHH preparation), they display unique genomic features, including their absence from promoter regions, and enrichment in CpG-poor intronic sequences.

### Functional significance of DNA methylation changes upon HBV infection

**Figure 5:**
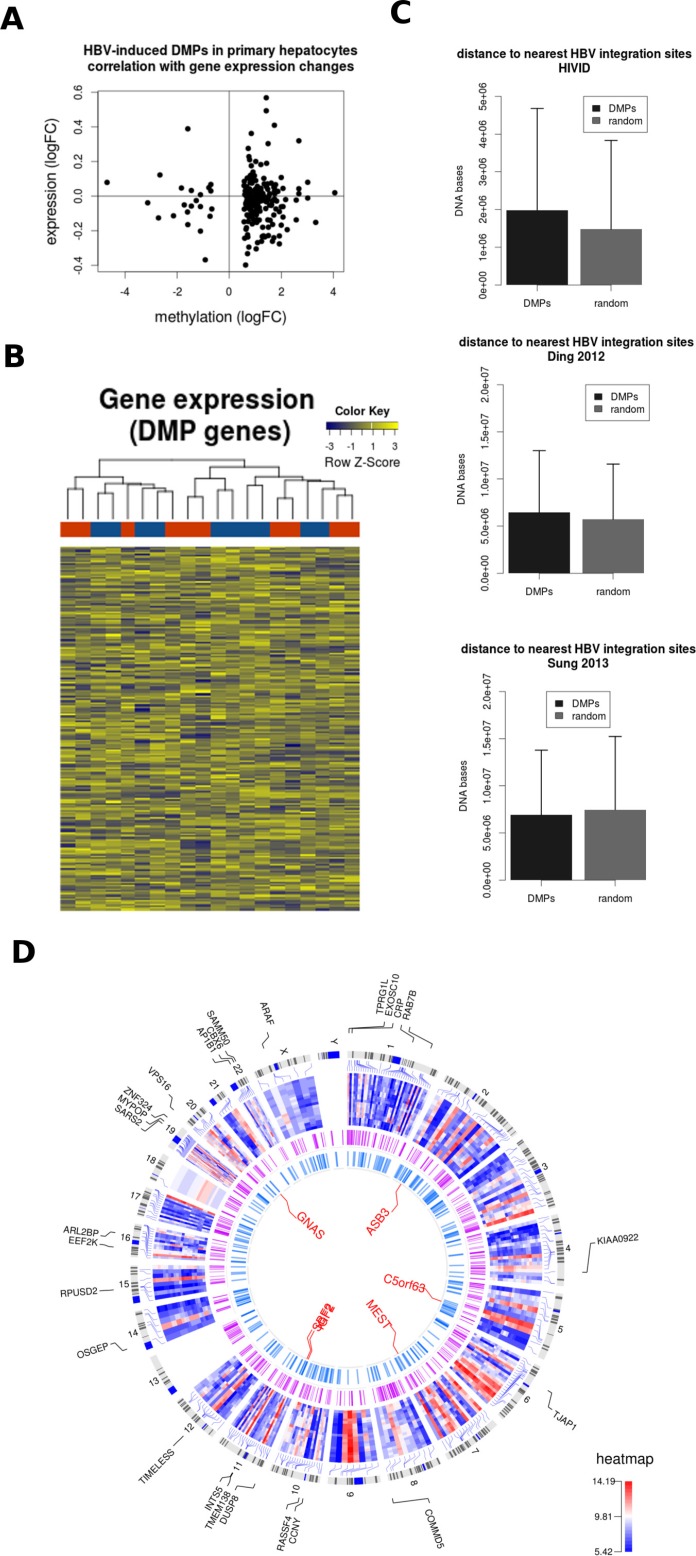
HBV DNA methylation signature and HBV integration sites (**A**) Gene expression data corresponding to HBV-related differentially methylated positions (DMPs) was extracted for mock and HBV conditions. The mean log expression and methylation data are shown in a correlation plot. (**B**) Expression data corresponding to the same selection of DMPs (those common across all time points) is shown in a heatmap representation. The unsupervised analysis was not able to discriminate between mock (blue) and HBV (red) conditions. (**C**) The genomic distance between DMPs and HBV integration sites was calculated for 3 published datasets (i.e. Xu et al 2013, Ding et al 2012, and Sung et al 2012). (**D**) Circos plot representation of the datasets analyzed in the present study. The external plot (blue-red heatmap) corresponds to the expression levels of differentially expressed genes in all mock vs HBV comparisons. Gene symbols corresponding to those genes differentially expressed across all time points are also shown. The intermediate circle illustrates HBV integration sites (combined integration sites from the 3 datasets used in [C]). The inner plot shows the common DMPs and the names corresponding to the top DMRs in the mock vs. HBV comparisons (i.e. *GNAS, ASB3, C5orf63, MEST, SCF2, and IGF2*).

A well-known function of DNA methylation is the regulation of gene expression. This is especially true at the level of promoter DNA methylation [1], although less is known about the impact of DNA methylation variation in other genomic locations. When comparing differentially expressed and differentially methylated genes induced by HBV, we did not find a significant overlap (data not shown), despite both DNA and RNA used for genome-wide analyses being obtained from the same PHH donor. In a more targeted analysis, we extracted the gene expression data for all DMPs induced by HBV throughout the time course experiment. Also in this analysis, no clear correlation was observed between DNA methylation and gene expression changes (Figure 5A). Indeed, gene expression data corresponding to DMP-associated genes was not able to discriminate between mock- and HBV- infected PHH (Figure 5B). This lack of global correlation may be related to the genomic distribution of DMPs, outside of gene promoters. However, we cannot rule out an impact on gene expression at specific loci.

Finally, we studied the potential link between DNA methylation and HBV genomic integration. Several studies have defined hot spots for HBV integration, frequently associated to liver cancer [20–22]. We calculated the genomic distance between DMPs and three reference HBV-integration datasets. In two of them (Xu et al 2013 and Ding et al 2012), DMPs were slightly more distant to HBV integration sites compared to a randomly generated list of genomic locations (Figure 5C), although these differences did not reach statistical significance.

Overall, no clear association was observed between gene expression and DNA methylation in HBV-infected PHH. A potential association between DNA methylation and HBV integration would require further studies.

## DISCUSSION

It has been proposed that DNA methylation links risk factor exposures to cellular phenotype. We illustrate this concept here by showing the genomic consequences of HBV infection on host primary human hepatocytes.

As a model, *ex vivo* culture of PHH is limited by the known loss of differentiation and the induction of cell death after few weeks. On the other hand, the use of immortalized hepatocyte-like cell lines carries its own systematic problems, including the loss of a number of differentiation features [13]. Therefore, we considered several factors to better mimic the *in vivo* infection by HBV. These factors include the use of natural infection with HBV virions, as opposed to transduction of the viral genome (e.g. by using baculovirus infection), the use of relevant “mock” controls exposed to the same supernatant after removal of viral particles, the use of controls at all time points, and the limitation of the infection to relatively early infection (i.e. up to 12 days) to minimize the effect of cell death.

Gene expression changes were shown to be highly dynamic and cumulative across the time-course experiment, independently of HBV infection. The enrichment in HNF4A targets, a master transcription factor of hepatocyte cell fate, suggests that a process of dedifferentiation is taking place. Our characterization of this process is relevant for those using similar models of *ex vivo* cell culture of human hepatocytes. Indeed, in similar models using primary cell lines we would recommend the use of controls at each time point of culture. Using such strategy we were able to derive a signature of HBV infection. This signature was enriched in Interferon Type I signaling pathway genes at early time points, an expected innate response to viral infection. It should also be considered that a small fraction of immune cells (e.g. Kupffer cells) are still present in the PHH preparation at the moment of nucleic acids extraction and may be responsible for one fraction of the observed responses to HBV infection. At later time points, HBV-dependent changes were enriched in several metabolic pathways. This last finding may be also linked to the differentiation status of PHH, although it was independent from the effect of cell culture conditions. From all time points of this analysis, the 24 hours data produced the most unique profile of expression, matching the expected peak of HBV infection. Within the genes deregulated at this time point, the DNA methyl-transferase cofactor *DNMT3L* was especially relevant to the DNA methylation changes induced by HBV. DNMT3L is a fundamental cofactor for the two *de novo* DNA-methyl-transferases DNMT3A and DNMT3B, and has been implicated in the control of gene imprinting (i.e. genes mono-allelically expressed in a parent-of-origin fashion).

The finding of *DNMT3L* downregulation goes in line with the presence of several DMRs on known imprinted genes (e.g. *MEST* and *GNAS*) after HBV infection, and their deregulation in HCC previously reported [23, 24]. At the site-specific level, we also observed consistent changes of DNA methylation across all experimental time points. Several of these DMPs were annotated to genes involved in gap junction and axon guidance, and are potentially linked to cell differentiation. However, the most striking finding about this methylation signature was its genomic distribution. DMPs tended to be absent from promoter CpG islands. Instead, DMPs were enriched in CG-poor intronic regions, far from transcription start sites. This supports recent genome-wide studies showing that methylome-wide variation may concentrate in intragenic non-promoter regions in different settings [25, 26]. In line with our findings, it has been recently shown that HBx is able to induce hypomethylation of distal intragenic CpG islands linked to the downregulation of DNMT3L and DNMT3A [27].

In general, gene expression does not seem to be directly associated with HBV-induced changes in DNA methylation. In addition, we found no correlation between the number of differentially expressed genes and the time of HBV infection (or the level of expression of viral proteins, as shown in Figure S1A and S1B). The power to detect differential expression at a given time point may be influenced by technical issues such as the signal/noise ratio of a specific sample and, therefore, the variation of the data. Therefore, the expression changes observed in PHH are the sum of background expression changes due to cell culture conditions and direct and indirect effects of viral proteins.

In addition, we did not observe a clear link between DMPs and known sites of HBV genomic integration. Instead, each layer of genomic regulation seems to target independent locations (Figure 5D). Therefore, other possibilities such as chromatin states, enhancer activity, or transcription factor binding sites, need to be considered when trying to understand the role of DNA methylation in non-promoter regions. Regardless of this, it was interesting to find “calcium signaling by hbx of hepatitis b virus” within the significant pathways enriched in HBV-induced DMPs, represented by 2 differentially methylated genes (i.e. *SRC* and *CREB1*). The HBV HBx protein is essential for viral replication in liver cells (a process enhanced by Src proto-oncogene activation [28] and it is believed to enter the nucleus to act as a transcriptional regulator. Two independent evidences are combined in this pathway: 1. HBx interacts with the transcription factor CREB and increases its DNA-binding activity [29], and 2. HBx may increase calcium release into the cytoplasm, affecting different signaling pathways [30]. However, a potential control of SRC and CREB expression by DNA methylation has not been reported.

Finally, variability between PHH donors is an important factor to consider. In our study, differential expression was correctly validated in an independent PHH infection, but this was not the case for methylation data (although technical validation in the same PHH donor was adequate). This variability may reflect the phenotypic heterogeneity of metabolically active players in human hepatocytes, such as cytochrome family genes. In addition, it may reflect differences between donors (e.g. age, sex) or differential susceptibility to viral infection.

In summary, this is to our knowledge the first report on the ability of HBV to induce genome-wide DNA methylation changes after natural infection of primary human hepatocytes. Although the bead array technology used here covers only a fraction of potential methylation sites in the human genome, we were able to show non-random and stable effects of HBV at specific genomic locations. Further studies are required to understand the consequences of these changes, and their potential use as biomarkers of HBV infection.

## MATERIALS AND METHODS

### Ethics statement

The use of human hepatic specimens for scientific purposes has been approved by the French National Ethic Committee.

### Cell culture and treatments

Primary human hepatocytes (PHH) were prepared from adult patients undergoing lobectomy or segmental liver resection for liver metastasis at the Centre Leon Berard (Lyon, France) with informed consent. PHH were isolated from non-tumoral tissue of surgical liver resections, and were cultured and infected with HBV as previously described [31–33]. HBV inocula were prepared as described [34]. Briefly, HBV was concentrated from the supernatant of HepG2.2.15 cells using centrifugal filter devices and tittered by HBV-DNA dot blot analysis after sedimentation into a CsCl density-gradient to determine enveloped DNA-containing viral particles. PHH were infected at a multiplicity of infection (MOI) of 1000 pfu/cell, with an estimated efficiency of at least 50%. Infected PHH and corresponding controls were kept for 4 hours, 8 hours, and 1, 6, and 12 days. Supernatants were obtained to validate the efficiency of infection by ELISA (Figure S1B), and nucleic acids were extracted for expression and DNA methylation analyses.

### Bisulfite modification and pyrosequencing

After trypsinization, cells were pelleted and resuspended in lysis buffer (1% SDS, 0.1 M NaCl, 0.1 M EDTA, 0.05 M Tris pH8) with Proteinase K (500 ug/ml) and incubated for 2 to 3 hours at 55°C. DNA was saturated with NaCl (6 M), precipitated with isopropanol, and cleaned with 70% ethanol. Extracted DNA was finally resuspended in water. Quantity and quality of the extracted DNA were assessed with a ND-8000 spectrophotometer (Nanodrop, Thermo scientific). To quantify the percentage of methylated cytosine in individual CpG sites, we performed bisulfite pyrosequencing, as previously described [11]. For samples processed for Infinium bead arrays, the conversion was performed on 600 ng of DNA using the EZ DNA methylation Kit (Zymo Research) and modified DNA was eluted in 16 ul of water. Quality of modification was checked by PCR using modified and unmodified primers for *GAPDH* gene. Pyrosequencing assays (primers for PCR, sequencing primers and regions) are described in Table S1.

### Bead array methylation assays

Methylation profiles of the different samples were analyzed using the 450K Infinium methylation bead arrays (Illumina, San Diego, USA). Briefly, the Infinium Humanmethylation450 beadchip interrogates more than 480,000 methylation sites [35]. The analysis on the bead array was conducted following the recommended protocols for amplification, labeling, hybridization and scanning. Each methylation analysis was performed in triplicate.

### Whole genome expression array

Total RNA was isolated using the TRIzol Reagent (Invitrogen) according to the manufacturer's instructions. RNA quantity and quality were assessed with a ND-8000 spectrophotometer and bioanalyzer. 500 ng of total RNA was used for each Human HT-12 Expression BeadChips (Illumina), as previously described [36]. Four candidate genes were selected for validation using quantitative RT-PCR. The housekeeping gene *HPRT1* was used as internal control.

### Bioinformatics analysis

Raw methylation data was imported and processed using R/Bioconductor packages [37, 38]. Data quality was inspected using boxplots for the distribution of methylated and unmethylated signals, and inter-sample relationship using multidimensional scaling plots and unsupervised clustering. Probes were filtered for low quality (detection *P* value > 0.05) and known cross-reactive probes [39]. After removing one bad quality sample, the remaining dataset was background substracted, normalized using intra-array beta-mixture quantile normalization [40], and batch-corrected using the ComBat function of the “sva” package [41]. Methylation beta values were logarithmically transformed to M values before parametric statistical analyses, as recommended [42]. To define differentially methylated positions (DMPs), we modeled the study variables (i.e. HBV infection and time of culture) in a linear regression using an empirical Bayesian approach [43]. DMPs were selected based on a threshold for the adjusted *P* value (False Discovery Rate, FDR) of 0.05 and a differential methylation (delta beta) of at least 10%. Differentially methylated regions (DMRs) were identified with the bump hunting method using the recommended proximity-based criteria [44]. A DMR was defined by the presence of at least 2 differentially methylated CpG sites with a maximum gap of 500 bp. To define the enrichment for different genomic features we used a genomic range of DMPs and a randomly selected list to match against previously described annotations [45], and known HBV integration sites [20–22].

For gene expression analyses, raw bead array data was exported from Genome Studio (version 2010.3, Illumina) into BRB-ArrayTools software (version 4.3.1, developed by Dr. Richard Simon and the BRB-ArrayTools Development Team). Data was normalized and annotated using the R/Bioconductor package “lumi” [37]. Class comparison between groups of bead arrays was done computing a *t*-test separately for each gene using the log-transformed expression values. Only those probes with FDR < 0.05 were considered significant. Differentially methylated and expressed genes were further analyzed to determine functional pathways and ontology enrichment using Enrichr [46]. In light of the non-proportional representation of gene symbols within the 450 k array [19], we performed a Bonferroni correction of the raw methylation *P* values to adjust for the number of probes in the corresponding gene. Then, for each gene we selected the probe with the minimum Bonferroni-corrected P, and *P* values were further adjusted for the number of gene symbols on the array. Those genes with an FDR-adjusted *P* < 0.05 were taken for further pathway analyses, using Enrichr.

All expression and methylation data have been deposited to the Gene Expression Omnibus repository (GEO accession number GSE72068).

### Statistical analysis

R/Bioconductor packages were used for bead array analyses, as described above. For other comparisons, means and differences of the means with 95% confidence intervals were obtained using GraphPad Prism (GraphPad Software Inc.). Two-tailed student *t* test was used for unpaired analysis comparing average expression between classes. *P* values < 0.05 were considered statistically significant.

## SUPPLEMENTARY MATERIALS FIGURES AND TABLES



## Abbreviations

HBV: hepatitis B virus
CGI: CpG island
DMP: differentially methylated position
DMR: differentially methylated region
HCC: hepatocellular carcinoma
PHH: primary human hepatocytes.
