# Supplementary material for: Genomic responses to hepatitis B virus (HBV) infection in primary human hepatocytes
Source: Oncotarget. 2015 Nov 2;6(42):44877–91. doi: 10.18632/oncotarget.6270 (PMC4792598; doi:10.18632/oncotarget.6270)
Supplement: Supplementary file 1 [file oncotarget-06-44877-s001.pdf]

Genomic responses to Hepatitis B virus (HBV) infection in primary human hepatocytes

Supplementary Materials

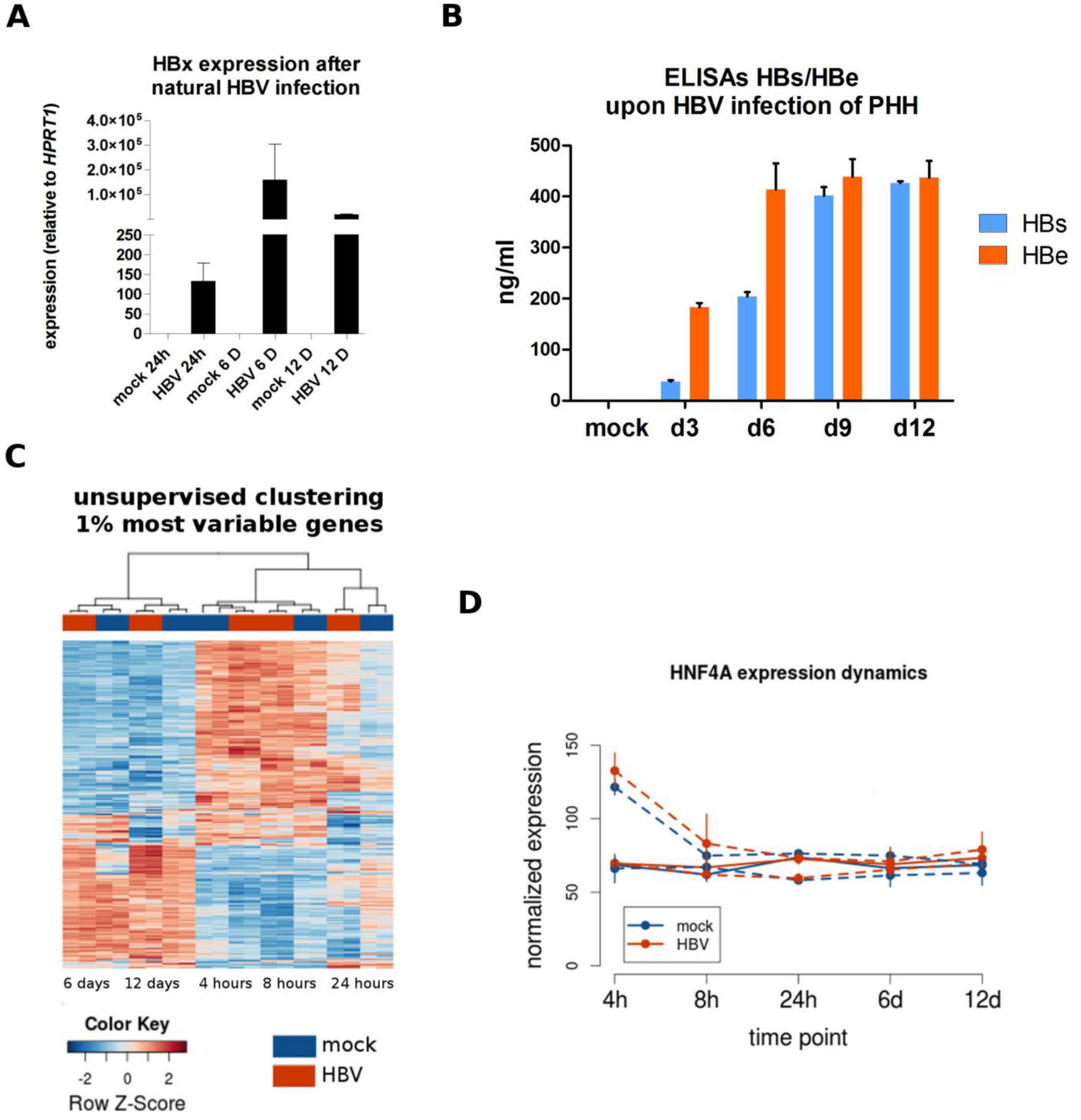

Supplementary Figure S1: Genomic responses to HBV infection in PHH. Primary human hepatocytes were naturally infected with HBV. Efficiency was monitored by qRT-PCR of HBx transcript (**A**) and ELISA for HB S and E proteins (**B**), at different time points. (**C**) Unsupervised clustering of the 1% most variable genes in PHH naturally infected with HBV during different time points and processed for whole genome expression using Illumina bead arrays. (**D**) Gene expression for the master transcription factor *HNF4A* was extracted from whole genome expression data, and their means for each time point were plotted separately for mock- and HBV-infected PHH. Three independent probes are represented.

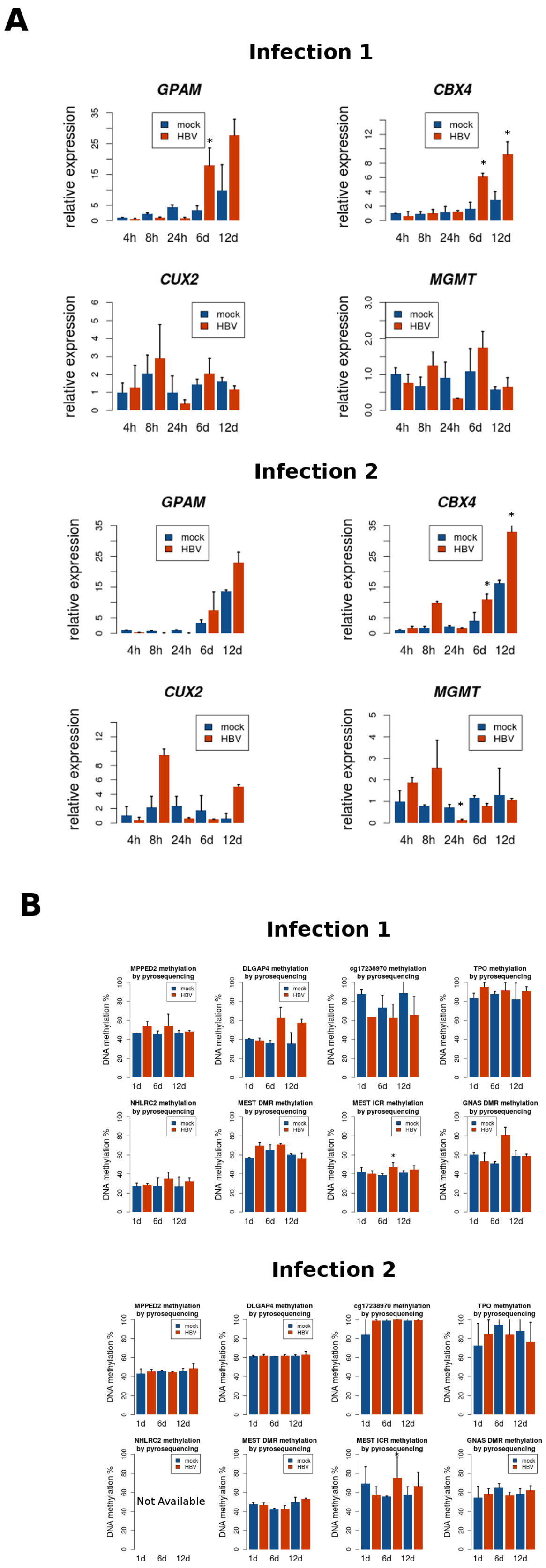

Supplementary Figure S2: Genomic responses to HBV infection in PHH. (**A**) Validation of selected differentially expressed genes (mock vs. HBV) using qRT-PCR, in two PHH samples from independent donors. (**B**) Validation of selected DMPs and DMRs (mock vs. HBV) using bisulfite pyrosequencing, in two PHH samples from independent donors.

Supplementary Table 1: Pyrosequencing assays

Genes	Refseq	UCSC localization	PCR primers	Sequencing primers	Infinium 450 k probe
DLGAP4	NM_014902	chr20:35062904-35062904	Fwd:GCCACTACT ACACGACCATCC Rev:[Btn]CAAACTTG GTTCGGAAGTCAT	GCCACTACTAC ACGACCATCC	cg02209770
TPO	NM_001206744	chr2:1,544,352-1,544,352	Fwd: GAAGTGTGAA ATGAAAGTGGG Rev:[Btn]CCAAATC CTATCCAACCACTA	ATAATTGGATA	cg06574769
cg17238970		chr1:81,124, 442-81,124,442	Fwd: TATGGTTTT GGGATGAG Rev:[Btn]CCCAAC ACTTTTCAAACT	TATGGTTTTG GGATGAG	cg17238970
NHLRC2	NM_198514	chr10:115,618, 670-115,618,670	For:TGGAAGAGTGG TTGATTTATTAGT Rev:[Btn]CATTCCACTA CAATTCATATCATTC	GTATATGGTTT	cg22819824
MPPED2	NM_001584	chr11:30,605, 525-30,605,637	Fwd:GTTTTGGYGGGA TTTTGGAATTTG Rev:[Btn]CCTCCCCCA AAAAAACCC	TGATATTTGGGAG	cg16820616

Supplementary Table 2: Transcriptome and methylome changes after HBV infection

Name	Description	Comment
DE mock all	differentially expressed genes in all mock conditions relative to mock 4 hours	FDR < 0.05, logFC > 1
DE mock 4 hvs 8 h	differentially expressed genes mock conditions 4 hours vs. 8 h	FDR < 0.05, logFC > 1
DE mock 4 hvs 24 h	differentially expressed genes mock conditions 4 hours vs. 24 h	FDR < 0.05, logFC > 1
DE mock 4 hvs 6 d	differentially expressed genes mock conditions 4 hours vs. 6 d	FDR < 0.05, logFC > 1
DE mock 4 hvs 12 d	differentially expressed genes mock conditions 4 hours vs. 12 d	FDR < 0.05, logFC > 1
DE all	differentially expressed genes HBV vs. mock across all time points	FDR < 0.05
DE 4 h	differentially expressed genes HBV vs. mock 4 hours	FDR < 0.05, logFC > 1
DE 8 h	differentially expressed genes HBV vs. mock 8 hours	FDR < 0.05, logFC > 1
DE 24 h	differentially expressed genes HBV vs. mock 24 hours	FDR < 0.05, logFC > 1
DE 6 d	differentially expressed genes HBV vs. mock day 6	FDR < 0.05, logFC > 1
DE 12 d	differentially expressed genes HBV vs. mock day 12	FDR < 0.05, logFC > 1
DE_all_symbols	summary HBV vs. mock comparisons	unique gene symbols used for pathway analysis
DE_pathways	top results of pathway analysis for expression	using Enrichr
DMPs_delta10	differentially methylation positions HBV vs. mock across all time points	FDR < 0.05, minimum change in methylation (delta_beta) of 10%
DMRs_all	differentially methylation regions HBV vs. mock across all time points	Top 100 regions (only FWER < 0.05 was considered significant)
DMRs_d1	differentially methylation regions HBV vs. mock at day 1	Top 100 regions (only FWER < 0.05 was considered significant)
DMRs_d6	differentially methylation regions HBV vs. mock at day 6	Top 100 regions (only FWER < 0.05 was considered significant)
DMRs_d12	differentially methylation regions HBV vs. mock at day 12	Top 100 regions (only FWER < 0.05 was considered significant)
DMPs_symbols	differentially methylated positions after adjusting for the number of probes in the HM450 bead array	gene symbols used for pathway analysis
DMPs_pathways	top results of pathway analysis for methylation	using Enrichr
